# SARS-CoV-2 Aerosol Exhaled by Experimentally Infected Cynomolgus Monkeys

**DOI:** 10.3201/eid2707.203948

**Published:** 2021-07

**Authors:** Chunmao Zhang, Zhendong Guo, Zongzheng Zhao, Tiecheng Wang, Liang Li, Faming Miao, Cheng Zhang, Yuanguo Li, Yuwei Gao

**Affiliations:** College of Veterinary Medicine at Hebei Agricultural University, Baoding, China. (C. Zhang);; Military Veterinary Research Institute, Changchun, China (C. Zhang, Z. Guo, Z. Zhao, T. Wang, L. Li, F. Miao, C. Zhang, Y. Li, Y. Gao)

**Keywords:** 2019 novel coronavirus disease, coronavirus disease, COVID-19, severe acute respiratory syndrome coronavirus 2, SARS-CoV-2, viruses, respiratory infections, zoonoses, cynomolgus monkeys, breath, size distribution, aerosol transmission

## Abstract

We analyzed size of severe acute respiratory coronavirus 2 (SARS-CoV-2) aerosol particles shed by experimentally infected cynomolgus monkeys. Most exhaled particles were small, and virus was mainly released early during infection. By postinfection day 6, no virus was detected in breath, but air in the isolator contained large quantities of aerosolized virus.

Although airborne transmission of severe acute respiratory syndrome coronavirus 2 (SARS-CoV-2) has been proven possible among humans (*1*), cats (*2*), ferrets (*3*), and Syrian hamsters (*4*), the relative roles of droplets and aerosols in the airborne transmission of SARS-CoV-2 remain controversial. A recent study showed that coronavirus disease (COVID-19) patients exhaled millions of SARS-CoV-2 particles during early infection stages (*5*). However, the size distribution of SARS-CoV-2 aerosol particles in exhaled breath of COVID-19 patients is not clear. 

To analyze size distribution of SARS-CoV-2 aerosols shed by cynomolgus monkeys, we inoculated 3 monkeys with SARS-CoV-2 via a combination of intranasal, intratracheal, and ocular routes. Monkeys were kept in individual cages placed in an isolator (biosafety housing with HEPA filters and independent ventilation system). The exhaled breath and air in the isolator were collected by a 6-stage Andersen sampler (https://tisch-env.com) at postinfection days 2, 4, and 6, and we quantified the viral RNA copies in samples (Appendix). We also determined size distribution of SARS-CoV-2 particles.

The virus particles the monkeys exhaled peaked at postinfection day 2 and ranged from 11,578 to 28,336 RNA copies during a 40-minute period. On average, each monkey exhaled 503 virus particles/min and 209.5 virus particles/L of exhaled breath. At postinfection day 4, the number of exhaled virus particles decreased substantially, ranging from 3,369 to 5,134 RNA copies during a 40-minute period. On average, each monkey exhaled 106 virus particles/min and 44 virus particles/L of breath. At postinfection day 6, no viral RNA was detected in exhaled breath (Figure, panel A; Appendix Figure 1). At postinfection days 2, 4, and 6, viral RNA was detected in air within the isolator housing the monkeys; we detected 6,182–13,608 RNA copies during a 30-minute period (Figure, panel C).

**Figure:**
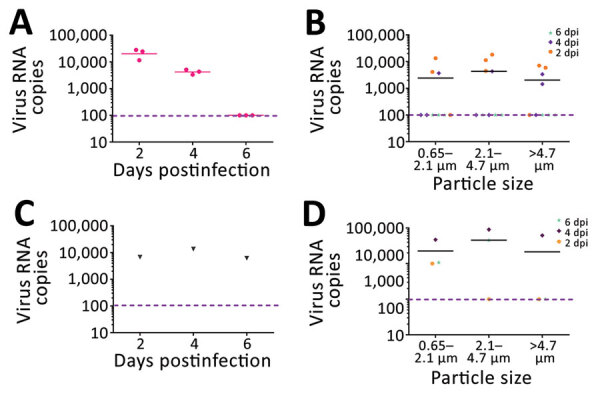
Viral RNA copies and size distribution of severe acute respiratory syndrome coronavirus 2 aerosols shed by experimentally infected cynomolgus monkeys. A) Viral RNA copies in aerosols directly expelled during 40 minutes of breathing. B) Size distribution of virus aerosols directly expelled during 40 minutes of breathing. C) Viral RNA copies in aerosols from the housing isolator during 30 minutes of sampling. D) Size distribution of virus aerosols in the isolator during 30 minutes of sampling. dpi, days postinfection. The pink dotted line indicates the limit of detection.

We measured size distribution of SARS-CoV-2 aerosol particles shed by the monkeys. In exhaled breath of inoculated monkeys and in air in the isolator, viral RNA was detected in all size bins, 0.65–2.1 mm, 2.1–4.7 mm, and >4.7 mm, at postinfection days 2 and 4; most were concentrated in the 2.1–4.7-mm bin (Figure, panels B, D; Appendix Tables 1, 2). For exhaled breath, virus particles in each of the 3 size bins accounted for 27.4%, 49.6%, and 23.0% of the total virus copies/40 min, respectively (Appendix Figure 3); for air in the isolator, virus particles in each of the 3 size bins accounted for 3.8%, 75.0%, and 21.2% of the total virus copies/30 min, respectively (Appendix Tables 1, 2, Figure 3). Most virus particles were in the smaller particle size range (0.65–4.7 mm), accounting for 77% to 79% of the total virus particles shed by the monkeys; droplets (>4.7 mm) accounted for »21%– 23% (Appendix Tables 1, 2, Figure 3). We tried to isolate live virus by sequentially passaging these samples in Vero-E6 cells 3 times (Appendix) but obtained no live virus and observed no cytopathic effects; the reasons for this failure are unknown. 

The World Health Organization cites the 2 main transmission routes of SARS-CoV-2 as large respiratory droplets and contact transmission. However, we found that monkeys infected with SARS-CoV-2 emitted large quantities of virus aerosol particles, most of which were smaller (<4.7 μm). Ma et al. showed that COVID-19 patients exhaled millions of SARS-CoV-2 particles/hour (*5*), far more than that noted for monkeys. This variation may result from biological differences between humans and monkeys and different sampling methods. Respiration is much slower in monkeys (2.4 L/min) than in humans (12 L/min). In addition, during sampling, monkeys were anesthetized and breathed slowly through their nostrils, possibly emitting fewer virus particles than when awake. The size of airborne particles determines how the virus is transmitted. Droplets (>4.7 μm) can travel limited distances; smaller particles (<4.7 μm) stay airborne longer and spread widely (*6*,*7*). Our findings suggest that aerosol transmission might contribute to SARS-CoV-2 spread. Personal protection requires wearing face masks, maintaining social distancing, and reducing gatherings. Infection risk in enclosed spaces is lowered by natural wind or mechanical airflow ventilation.

Cynomolgus monkeys infected with SARS-CoV-2 emitted most virus particles in early infection stages; particles decreased substantially at postinfection day 6. Zhou et al. demonstrated that COVID-19 patients emitted fewer virus particles when they were recovering and ready for discharge than did those in early infection stages (*8*). At postinfection day 6, no virus was detected in the breath of monkeys, but air in the isolator housing the monkeys still contained large quantities of aerosolized virus. These different seemingly noncoherent observations can be attributed to monkey activity, air flow, and some virus aerosol residues exhaled by monkeys for a relatively long period before sampling. Recently, Asadi et al. showed that aerosolized fomites (microscopic particles) played a role in influenza virus transmission between guinea pigs (*9*). SARS-CoV-2 may be carried and transmitted between humans by aerosolized fomites. Most SARS-CoV-2 aerosol particles exhaled by the cynomolgus monkeys in this study were smaller, suggesting that aerosols might be a route for SARS-CoV-2 transmission.

Appendix Supplemental methods and results for study of SARS-CoV-2 aerosol exhaled by cynomolgus monkeys.
