# Supplementary material for: SARS-CoV-2 Aerosol Exhaled by Experimentally Infected Cynomolgus Monkeys
Source: Emerg Infect Dis. 2021 Jul;27(7):1979–81. doi: 10.3201/eid2707.203948 (PMC8237865; doi:10.3201/eid2707.203948)
Supplement: Appendix — Supplemental methods and results for study of SARS-CoV-2 aerosol exhaled by cynomolgus monkeys. [file 20-3948-Techapp-s1.pdf]

Article DOI: https://doi.org/10.3201/eid2707.203948

SARS-CoV-2 Aerosol Exhaled by Experimentally Infected Cynomolgus Monkeys

Appendix

Methods

Virus and cells

Vero-E6 cells were grown in Dulbecco's modified Eagle's medium (DMEM) containing 10% feline bovine calf serum and 1% penicillin and streptomycin. The SARS-CoV-2, BetaCoV/Beijing/IME-BJ05-2020 (abbreviated as V34) was propagated in Vero-E6 cells maintained in DMEM containing 2% feline bovine calf serum and 1% penicillin and streptomycin. The viable SARS-CoV-2 was quantified by the classical 50% endpoint assay ($TCID_{50}$ assay) base on the virus-induced cytopathic effects (CPE).

Animals, ethics and biosafety statement

Three cynomolgus monkeys, aged 4 to 6 years old, were ordered from animal center of Academy of Military Medicine Science. Animals were housed in an negative pressured isolator with a humidity of 50% at 22°C. All animal experiments were approved by the Animal Care and Use Committee of the Military Veterinary Research Institute and carried out by certified staff. Food and water were available in ad libitum. All experiments involved with the infectious SARS-CoV-2 were performed in the Animal Biosafety Level 3 laboratories of the Military Veterinary Research Institute.

Sample collection and processing

For sampling exhaled breath of monkeys, three naive cynomolgus monkeys, sero negative and nucleic acid negative for SARS-CoV-2, were inoculated via a combination of intranasal (0.5ml each nostril), intratrachea (4ml) and ocular (200μl) routes with$10^{6.5}$ $TCID_{50}$/ml of SARS-CoV-2 virus dilution and housed separately in cages placed in the isolator. The sampling experiments were performed in a biosafety level 2 cabinet (Appendix Figure 2). A plastic box was put in the cabinet. The length, width and height of the box are 60 cm, 40 cm and 30 cm. On the upper left side wall of the box, a small hole was made as air inlet, whereas on the lower right side wall of the box, a small hole was made to link the 6-stage Andersen sampler (Tisch, America) to the air sampling pump (Qingdao Junray Intelligent Instrument Co Ltd, Qingdao, China). A foam pad was put in the box and the Andersen sampler was placed on the front right side in the box. Air sampling pump was placed on the right side of the box in the cabinet. The air sampling pump was linked to the Andersen sampler with a soft plastic tube through the small hole on the lower right side of the box. Gelatin filters were put into six plastic plate and installed into the Andersen sampler. Each monkey was anesthetized and kept in the plastic box, and the monkey faces the sampling port of the sampler. The distance between the head of the monkey and the sampling port is about 10 cm. The height of the animal head is about the same as the height of the sampling port of the Andersen sampler. Virus aerosol particles exhaled by the monkey were released into air in the box, and then were collected onto gelatine filters (Satorius, Germany) for 40 minutes at 2, 4 and 6 days post infection(dpi) using the 6-stage Andersen sampler that separate airborne particles into six ranges (0.65-1.1 μm, 1.1-2.1 μm, 2.1-3.3 μm, 3.3-4.7 μm, 4.7-7 μm, >7 μm) at a flow rate of 28.3 L/min.

As simulation of environment aerosols, like SARS-CoV-2 aerosols in a hospital ward, we also sampled air in the isolator housing the monkeys. When sampling air in the isolator, each monkey was housed in a separate cage placed in the isolator, and were not anaesthetized and can freely move in cages. The ventilation rate of the isolator is 20 times each hour. The Andersen

sampler was placed between two cages, and the distance between these two cages is about 25 cm. The air sampling pump was placed in front of the cages, and was linked to the Andersen sampler with a soft plastic tube. Air in the isolator containing aerosolized fomites (contaminates on the cage surface and fences in the cages) caused by animal activity and air flow, and virus aerosols exhaled by the monkeys was collected with this sampler for 30 minutes. The gelatine filters were dissolved in 5 mL normal saline and melted at 37℃ in water bath, and 500 μL of RNA lysis buffer was added to 500 μL of dissolved samples to inactivate the virus, and then temporarily stored in 4℃ for RNA extraction and quantification.

RNA extraction and quantification

RNA was extracted from samples in a specified lab for SARS-CoV-2 with the viral RNA minikits according to the manufacturer's protocol (QIAGEN, Hilden, Germany). Real-time PCR was used to quantify the viral RNA copies of SARS-CoV-2 in these samples with the Detection Kits for 2019-Novel Coronavirus RNA (Shenzhen Puruikang Biotech, China). The detection limit of this diagnostic kit is about 100 copies/mL. The amplification reaction was performed with ABI7500 system (Roche, Switzerland) and the reaction conditions were 50 °C for 30 min for reverse transcription, followed by 95 °C for 1 min and then 45 cycles of 95 °C for 15 s, 60 °C for 30 s. The numbers of the viral RNA copies in samples were estimated from the measured cycle threshold (Ct) values. A standard curve was fitted using a series of 10-fold dilutions of a standard plasmid DNA of SARS-CoV-2. The fitted standard curve was Ct = -3.44X0 + 41.02, where X0 is the initial viral RNA copies in the reaction system.

Virus concentration calculation in the breath

According to the body weight of these cynomolgus monkeys, we made an estimate of the respiratory volume per minute. We assumed that the average respiratory rate of cynomolgus monkeys is about 40 times per minute and the average respiratory volume each time is 60 mL. Hence, the respiratory volume per minute would be 2400 mL/min, that is 2.4 L/min. Then we

calculated the virus concentrations in the breath by dividing the total RNA copies by the total respiratory volume during a 40-minute sampling. The total respiratory volume was the product of sampling time and the respiratory volume of the monkeys per minute.

Virus isolation in Vero-E6 cells.

Virus isolation was performed in a BSL-3 laboratory. Cells grown as monlayers in 6-well plates were inoculated when they were as 80% confluency. The cell culture medium was removed, and 1 mL of the dissolved aerosol sample was centrifuged at 6000 rpm, and the supernatant was pipetted and added to the cell culture plates. After one hour incubation at 37°C in 5% CO2, 1 mL cell culture medium containing 2% fetal bovine calf serum and 2% penicillin and streptomycin was added to the wells. Mock-infected cell cultures were parallelly maintained. The cells were daily observed for cytopathic effect for 4 days. And viral RNA copies in the culture medium at 4 dpi was quantified as the methods in RNA extraction and quantification. The collected supernatant was used to sequentially infect Vero-E6 cells for two times. Cytopathic effect of the infected cells were daily observed and viral RNA copies in the culture medium was monitored as before. During the experimental period, if an obvious cytopathic effect was observed in the vero-E6 cells, and viral RNA copies in the supernatant obviously increased, then live virus was thought to be successfully obtained.

Appendix Table 1. Aerosol shedding of SARS-CoV-2 in infected cynomolgus monkeys*

	Virus RNA copies								
	Particle size (μm)								
Time	0.65-1.1	1.1-2.1	2.1-3.3	3.3-4.7	4.7-7	>7	0.65-2.1	2.1-4.7	>4.7
Day 2	9360	4085.3	3558.2	7785.6	-	-	13445.3	11343.8	-
	-	4180.8	-	18249.6	-	5905.4	4108.8	18249.6	5905.4
	-	-	-	4478.4	7099.2	-	-	4478.4	7099.2
Day 4	-	-	-	4341.6	-	-	-	4341.6	-
	3693.6	-	-	-	-	1440	3693.6	-	1440
	-	-	-	-	3369.2	-	-	-	3369.2
Day 6	-	-	-	-	-	-	-	-	-
	-	-	-	-	-	-	-	-	-
	-	-	-	-	-	-	-	-	-
total							21247.7	38413.4	17813.8
Ratio (%)							27.4	49.6	23.0

*-, not detected.

Appendix Table 2. The number of the viral RNA copies in the isolator*

	Virus RNA copies								
	Particle size (μm)								
Time	0.65-1.1	1.1-2.1	2.1-3.3	3.3-4.7	4.7-7	>7	0.65-2.1	2.1-4.7	>4.7
Day 2	-	1002.7	2826.2	1867.2	-	1074.7	1002.7	4693.4	1074.7
Day 4	-	-	-	9064.8	-	4543.2	-	9064.8	4543.2
Day 6	-	-	6182.4	-	-	-	-	6182.4	-
total							1002.7	19940.6	5617.9
Ratio (%)							3.8	75	21.2

*-, not detected.

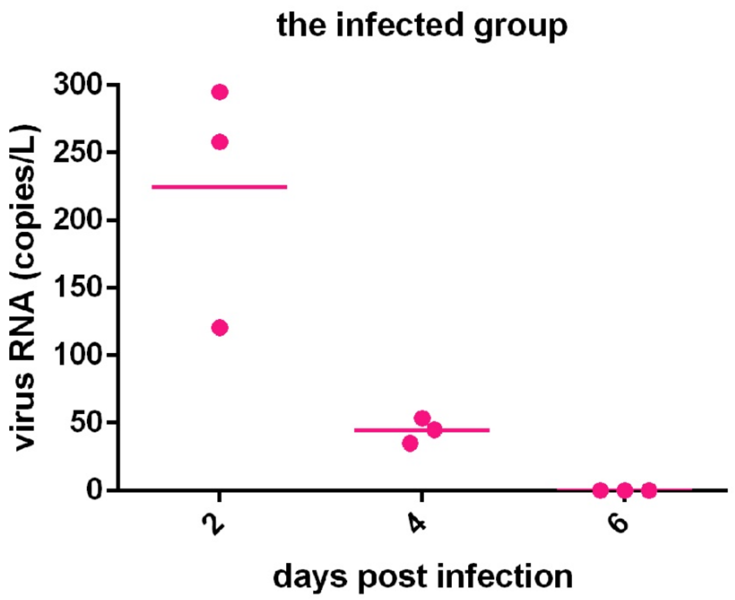

Appendix Figure 1. Aerosol shedding rate of SARS-CoV-2 in air exhaled by the infected monkeys.

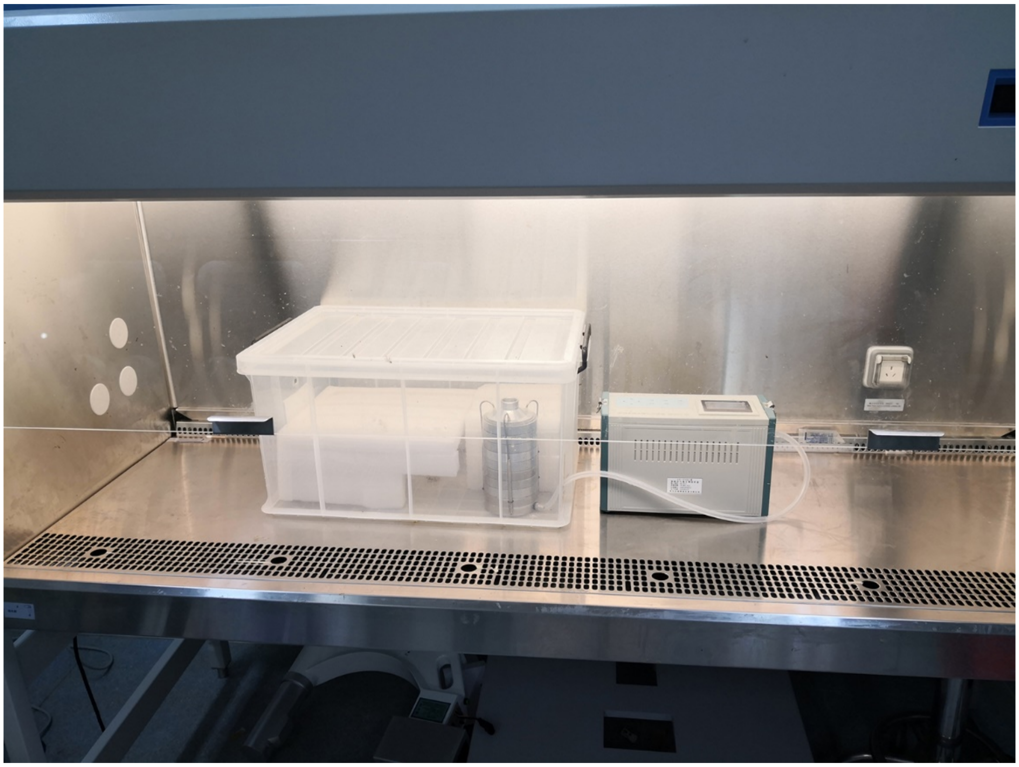

Appendix Figure 2. The experimental setup for aerosol sampling in exhaled breath by the monkeys.

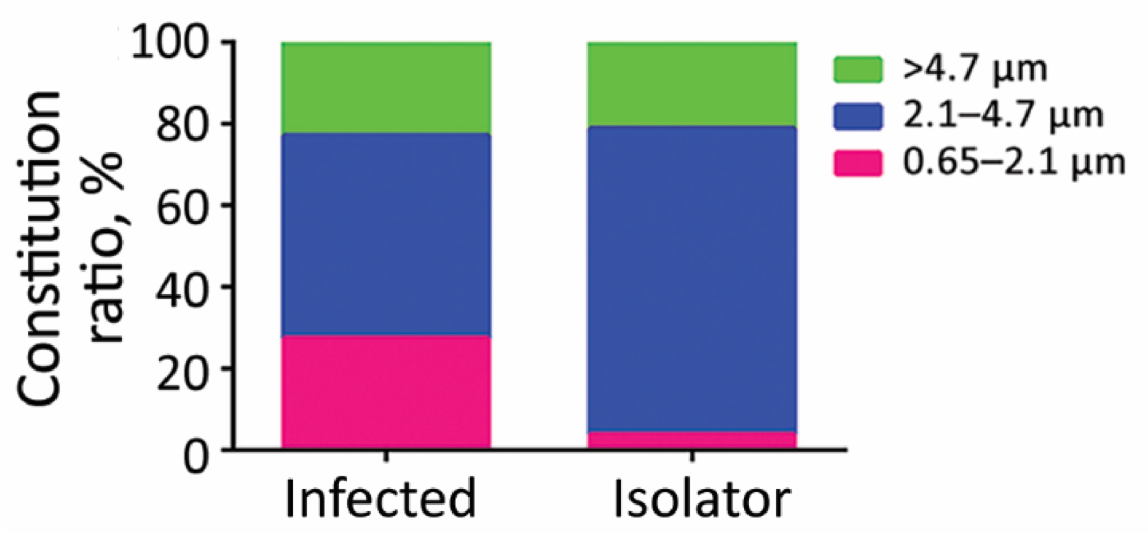

Appendix Figure 3. Percentage of virus particles in each of 3 size bins (constitution ratio) in study of severe acute respiratory syndrome coronavirus 2 aerosols shed by experimentally infected cynomolgus monkeys.
